# How do familiarity and relatedness influence mate choice in *Armadillidium vulgare*?

**DOI:** 10.1371/journal.pone.0209893

**Published:** 2018-12-31

**Authors:** Margot Fortin, Camille Vitet, Catherine Souty-Grosset, Freddie-Jeanne Richard

**Affiliations:** Laboratoire Ecologie et Biologie des Interactions, Team Ecologie, Evolution, Symbiose, Université de Poitiers, Poitiers, France; Universidade de São paulo, BRAZIL

## Abstract

Mate choice is an important process in sexual selection and usually prevents inbreeding depression in populations. In the terrestrial isopod *Armadillidium vulgare*, the close physical proximity between individuals may increase the risk of reproducing with siblings. Moreover, individuals of this species can be infected with the feminizing bacteria of *Wolbachia*, which influence male mate choice. However, little is known about the kinship or familiarity assessment of the selected partner that occurs when a male can choose between females with or without *Wolbachia*. To investigate the potential mechanisms leading to mate choice and the potential impact of the parasite, we performed behavioral choice tests on males where they could choose between sibling *vs*. nonsibling females, familiar *vs*. unfamiliar females, and sibling familiar *vs*. unfamiliar nonsibling females. To investigate the costs of inbreeding, we compared the reproductive success of both sibling and nonsibling mates. Our results revealed that male copulation attempts were higher for familiar females and for nonsibling females when both females were *Wolbachia*-infected, but the duration was longer when both females were *Wolbachia*-free. When males mated with a sibling female, their fecundity was severely decreased, consistent with inbreeding depression. Overall, we observed copulations with all types of females and demonstrated discrimination capacities and potential preferences. We highlight the complexity of the tradeoff between kinship, familiarity and parasite transmission assessment for mate choice.

## Introduction

Mate choice is defined as an individual preference for members of the opposite sex, and according to evolutionary theory, such choice evolves by sexual selection [1]. Mate choice is an important process as the genetic compatibility between sexual partners affects offspring viability and therefore the maintenance of the population through evolution. Many studies have shown that reproduction involving individuals with similar genotypes leads to an increase in homozygous deleterious mutations, which in turn induces a lower adaptability to changing environments and a decline in individual fitness. This effect is generally referred to as “inbreeding depression” [2, 3].

Inbreeding depression has been observed in many species including vertebrates (e.g., the red deer *Cervus elaphus* [4], and the black grouse *Tetrao tetrix* [5] and invertebrates (e.g., the cabbage beetle *Colaphellus bowringi* [6], and the parasitoid wasp *Venturia canescens* [7]). Some species have evolved inbreeding-avoidance mechanisms such as dispersal, especially when dispersing individuals are less likely to mate with kin and have higher fitness (e.g., the black grouse *Tetrao tetrix* [8], and the great tit *Parus major* [9]). However, in gregarious or social species, which typically are poor dispersers, kin recognition is another mechanism of avoiding inbreeding depression [10]. Kin recognition is defined as the capacity to recognize related individuals, which involves a detection of kinship based on congeners’ recognition mechanisms [11, 12].

Kin recognition can be based on familiarity, phenotype matching or allelic recognition [13, 14]. In the case of familiarity, animals learn to recognize the signature of congeners (i.e., siblings) during development and later discriminate these familiar individuals from unfamiliar ones [12]. In nesting species such as social insects, kin recognition is generally linked to nestmate recognition [15]. Phenotype matching does not necessarily imply the learning of siblings' labels but instead the comparison of an individual’s own phenotype with the phenotype of unknown individuals [16]. Allelic recognition is a genetic mechanism where an individual recognizes related individuals through allelic perception without any preliminary learning [17]. For example, single genomic elements marked by a protein-encoding gene (known as *Gp*-9) determine whether workers tolerate a single fertile queen or multiple queens per colony [18].

According to Parker [19], inbreeding avoidance mechanisms should be favored in an organism if both the costs associated with global investment in reproduction and the costs linked to inbreeding depression are high. For example, in the parasitoid wasp *Venturia canescens*, in which mating with siblings increases the risk of producing sterile or unviable offspring, females avoid mating with sibling males [20]. Conversely, individuals of species that can tolerate inbreeding do not show any inbreeding avoidance (e.g., the satin bowerbird *Ptilonorhynchus violaceus* [21], and the fruit fly *Drosophila melanogaster* [22]]. Inbreeding avoidance can also be the result of cryptic choice. For example, for the aphid *Acyrthosiphon pisum*, inbreeding induces costs in terms of the number of descendants and survival. Inbred males transfer sperm throughout copulation, but oviparae prevent the sperm from reaching their spermatheca. Therefore, inbreeding avoidance takes place between copulation and sperm transfer, suggesting potential cryptic female choice that could prevent constant male harassment [23].

Gregarious invertebrate species occupy a key range in the invertebrate social organization continuum and are pertinent models for studying mate choice as well as its underlying mechanisms. Indeed, the close physical proximity and interattraction between individuals increase the interaction and mating probabilities between males and females from the same group [24, 25]. In species where juveniles disperse, groups are composed of unrelated individuals; therefore, mating with individuals from the same group does not induce inbreeding depression [11]. In contrast, in species where the dispersal rate is low, mechanisms for kin recognition limit inbreeding between close relatives, such as the mechanism observed in the German cockroach *Blattella germanica* [10, 26].

In the terrestrial crustacean isopod *Armadillidium vulgare* (common woodlouse), individuals live in gregarious groups. Male mate choice occurs in this species, as males can discriminate between females from short distances based on chemical cues associated with their molting status [24]. A recent study showed that the female reproductive experience is also a factor affecting choice by males in this species, with virgin females being preferred over females that have had a previous reproductive experience [27]. However, whether discrimination based on kin recognition occurs remains unknown.

Parasitic infections are known to influence mate choice, and the avoidance of mating with infected mates often leads to an increase in fitness [28]. A relevant model for investigating the effect of bacterial infection on mate choice is the widespread alphaproteobacteria *Wolbachia*. These intracellular alphaproteobacteria are widespread, occurring in insects, arachnids, nematodes and crustaceans [29–32]. These proteobacteria increase their rate of transmission through the female germline, mainly by acting on the reproduction of their host. Their actions include cytoplasmic incompatibility, thelytokous parthenogenesis induction, male killing and feminization of genetic males [33]. A particularity of *A*. *vulgare* woodlice is that individuals can be parasitized by the *Wolbachia* bacteria. In *A*. *vulgare*, *Wolbachia* induce feminization of genetic males, transforming them into functional females [34, 35]. As a consequence, *Wolbachia* act as sex-ratio distorters in favor of females. *Wolbachia* are weakly virulent but may become pathogenic when the bacterial loads become too high [36]. It has been shown that the presence of *Wolbachia* has negative effects on *A*. *vulgare* females’ learning and memory capacities [37], survival [38], and attractiveness for mating [27, 39] and is probably linked to lower copulation investment [40]. Moreover, recent data indicate that males are able to discriminate *Wolbachia-*free females from *Wolbachia-*infected females from short distances and prefer the former type [27, 41]. Although infection can impact mate choice and the performance of infected individuals, little is known about its effect within the context of individual recognition used to assess kinship and familiarity.

The aim of this study was to assess whether individual recognition (kin and familiarity) and inbreeding avoidance take place in *A*. *vulgare* and, if so, to identify the underlying behavioral mechanisms. In addition, we sought to evaluate the possible impact of *Wolbachia* on kin recognition in this species. To identify a potential kin recognition mechanism, males were submitted to different encounters, allowing us to measure the effect of kinship, the effect of familiarity, and the combined effect of kinship and familiarity on mate choice. Males could choose between different kinds of females in two behavioral tests: (i) in a Y preference test, females' levels of attractiveness were compared; and (ii) in an open-field choice test, the preference of males between two types of females and the behavioral reaction of females to the males' courtship were investigated. Finally, to assess the potential cost of inbreeding, the number of descendants was compared between mate pairs composed of siblings (one male with one female from the same clutch) and nonsiblings (one male with one female from a different clutch). All tests were performed with both *Wolbachia-*free females and *Wolbachia-*infected females. We hypothesized that in *Wolbachia*-free females, males would prefer and mate more with unrelated females based on familiarity, leading to the adaptive avoidance of inbreeding. When males had to choose between two infected females, we expected no preference based on the relatedness and/or familiarity of females to enhance female reproductive success. Such a result was expected to be the consequence of *Wolbachia* altering the kinship discrimination phenotype and increasing the mating probability of infected females.

## Materials and methods

### Biological material and animal rearing

All *Armadillidium vulgare* individuals (Isopoda, Oniscidea) (Latreille, 1804) used in this study were from a population collected in Denmark. To obtain females infected by a feminizing *Wolbachia* strain (*w*VulC), part of the collected females were infected by *Wolbachia* in the laboratory [42], and bacteria were then naturally maternally transmitted to the next generation. Therefore, the offspring of these females, which were used in the present study, were naturally infected by *Wolbachia* (hereafter named “*Wolbachia-*infected”). All gravid females (with and without *Wolbachia*) were isolated, their offspring were sexed under a binocular microscope, and the males and females of these offspring were separated into different boxes before sexual maturity. The presence or absence of *Wolbachia* was tested on 10 randomly selected uninfected and naturally infected females by DNA extraction and PCR assays of dissected tissues (as in [41]). The animals were maintained under laboratory conditions at a temperature of 20°C (± 1°C), a humidity of 70% and the ambient photoperiod of Poitiers (France, 46°33’N and 0°22’E). The experiments were conducted in the spring. The reproduction of individuals was controlled in order to avoid inbreeding. Males and females were reared in moistened compost in separate compartments of the same boxes (17x11 cm; 15 individuals of each sex per box), with food provided *ad libitum* (dried lime leaves (*Tilia* sp.) and slices of fresh carrots). To maintain environmental familiarity between males and females, the boxes were divided into two equal parts with mesh allowing visual, olfactory and antennary contacts between males and females but preventing mating. Moreover, the positions of males and females in the boxes were switched every two weeks over the course of 4 months. Due to this switching, animals were allowed to access each other’s environment.

### Animal selection

Animals were all 1.5 years old and virgins. In this species, individuals undergo molting each month, and they are available for mating only during certain parts of their molting cycle [43]. During ecdysis, when they shed their cuticles, both males and females are vulnerable and unable to mate [43]. Moreover, the ovarian cycle is strongly linked to the molting cycle [44]. Females reach a peak in attractiveness at the beginning of the pre-ecdysis stage, a few days before the parturial molt [24], a stage that is recognizable by the appearance of characteristic white plates [45]. All females used for our experiments were at this stage in order to avoid differences in attractiveness among them due to differences in the period of the molting cycle [24]. Finding two females at the same molting cycle was a crucial step in the experiment and the main factor limiting replication. Males were used while in the intermolt stage, the stage during which they prefer to mate [43]. All individuals were marked with various colors on their cuticle (with a Posca marker) at least four hours before the behavioral experiments.

### Experimental groups

To test the effect of familiarity, males had the choice between a female reared in the same box (i.e., a familiar female) and a female reared in a different box (i.e., an unfamiliar female), both of which were unrelated to the male. To test the effect of kinship, males had the choice between a female from the same clutch (i.e., a sibling female) and a female from a different clutch (i.e., a nonsibling female), both of which were unfamiliar to the male. To test the effect of the interaction between kinship and familiarity, males had the choice between a female from the same clutch reared in the same box (i.e., a familiar sibling female) and a female from a different clutch reared in a different box (i.e., an unfamiliar nonsibling female). Finally, we tested male choice between a female from the same clutch but reared in a different box (i.e., an unfamiliar sibling female) and a female from a different clutch but reared in the same box (i.e., a familiar nonsibling female).

These encounters were tested with both *Wolbachia-*free females and *Wolbachia-*infected females using two behavioral tests.

### Behavioral tests

Behavioral experiments were conducted from March to June. To assess females’ attractiveness to males, Y preference tests were performed. To compare the interactions between males and the different kinds of females, open-field tests were performed. For each test, the device was covered with a transparent lid in order to limit airflow, and the inside of the Petri dish was covered with moistened filter paper renewed after each experiment. During both types of tests, the luminosity and the temperature were controlled (10 lux and 20°C).

#### Females attractiveness: Y preference test

**Fig 1:**
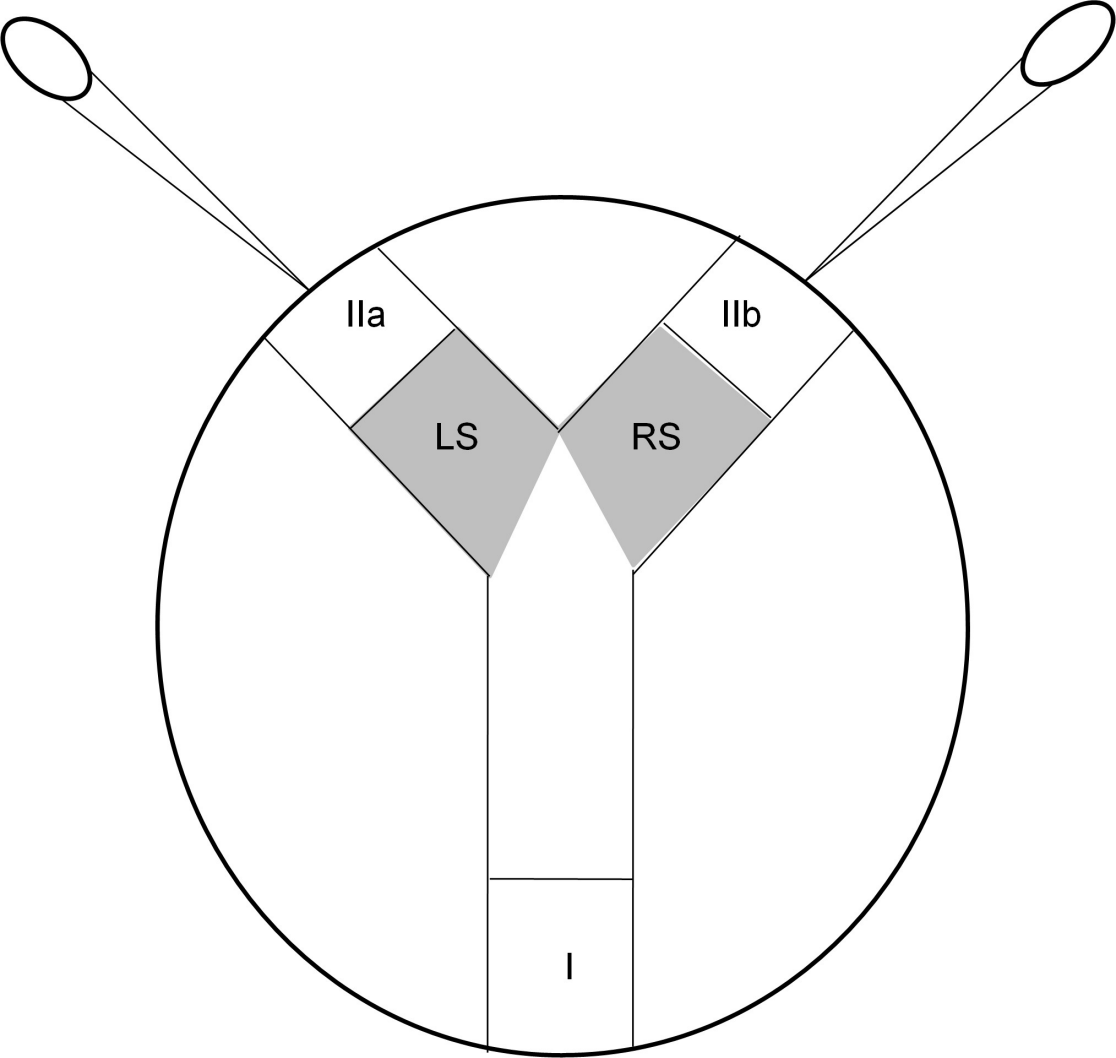
**Schematic view of the Y preference test used to investigate the preferences of males for different conspecifics in *A. vulgare*** (I: initial position of the tested male, IIa and IIb: positions of the target females, LS: left side; RS: right side). A plastic pipette was placed at the end of each of the two tunnels and used to pulse air regularly into the system; from [24]. (http://dx.doi.org/10.17504/protocols.io.vpfe5jn).

Tests were performed using a Y-shaped choice chamber built in a plastic Petri dish (diameter: 8.7 cm; height: 1.2 cm; lid included) (Fig 1, already set up by 24; http://dx.doi.org/10.17504/protocols.io.vpfe5jn). Rigid plastic tunnels were also used to create the device, and two plastic pipettes were sealed at the end of these tunnels to pulse air regularly into the system; the air was passed through sections (IIa) and (IIb) to spread the odor of the females. At the beginning of the experiment, the isopods were placed in the sections located in the three extreme parts of the tunnels: the two target females were placed in sections (IIa) and (IIb) (one in each section), and the tested male was placed in section (I). The male was then able to move into four other sections. The position of the target females in sections (IIa) and (IIb) was inverted between each replicate, and the target and tested individuals were used for only one test. The females were placed in their sections 15 minutes before the beginning of the test so that they could become accustomed to the new environment. The male was placed in section (I), and from the moment he entered the neutral section (NS), the time spent in the left section (LS) and the right section (RS) was monitored using the program EthoLog 2.2 (Ottoni, 2000). Each test lasted for 10 minutes. Male preference or female attractiveness was evaluated by comparing the time spent by the male in front of the section adjacent to the females (LS or LR). We compared males' preferences for familiar and unfamiliar females for both *Wolbachia-*free (N = 30) and *Wolbachia-*infected (N = 38) females and for sibling and nonsibling females for both *Wolbachia-*free (N = 38) and *Wolbachia-*infected (N = 30) females. Finally, we compared the attractiveness of familiar sibling females and unfamiliar nonsibling females for both *Wolbachia-*free (N = 34) and *Wolbachia-*infected (N = 35) females. To compare the attractiveness of unfamiliar sibling females and familiar nonsibling females, we prepared 10 boxes of 14 individuals (7 males and 7 females) for each interaction for two consecutive years, but we were able to obtain only 6 replicates (N_Wolbachia-free_) with the proper stage of synchronization for behavioral tests.

Individuals from the same clutch were allowed to interact in the maternal marsupium and separated before sexual maturity (approximately 4 months), even if they were considered unfamiliar siblings in the experiments.

#### Interactions between individuals: Open-field choice tests

To study the interactions between the males and the different kinds of females, open-field tests were performed in a Petri dish (diameter: 8 cm; height: 5 cm). The females were placed in the device 15 minutes before the beginning of the test. The male was then placed in the device, and individuals were able to interact with each other until the first successful copulation of the male. If neither female accepted the copulation attempts, the experiment was stopped after 4 hours. During the experiment, the copulation attempts were recorded and corresponded to long female body exploration by the male followed by mounting of the female’s dorsal surface. The occurrence and duration of male copulation attempts with both females were recorded.

The female's reaction to each copulation attempt (described in [46]] was also noted, and the five following behaviors were recorded: (1) Rolling and opening: volvation immediately followed by a slight opening, indicating that mating was accepted by the female (S1 Video); (2) Rolling without opening: volvation that did not allow the male to copulate with the female (S2 Video); (3) Escape: movement away from the male; (4) Immobilization: the female did not move until the male stopped his attempt; and (5) Topple: jolting movements during male mounting, which indicated that mating was refused by the female.

Finally, when a male performed copulation, we noted which female was chosen.

To minimize observer bias, a neutral observer analyzed the videos. The observer recorded all information based on an individual’s colors without knowing the meaning of the colors (female identity or experiment history).

**Fig 2:**
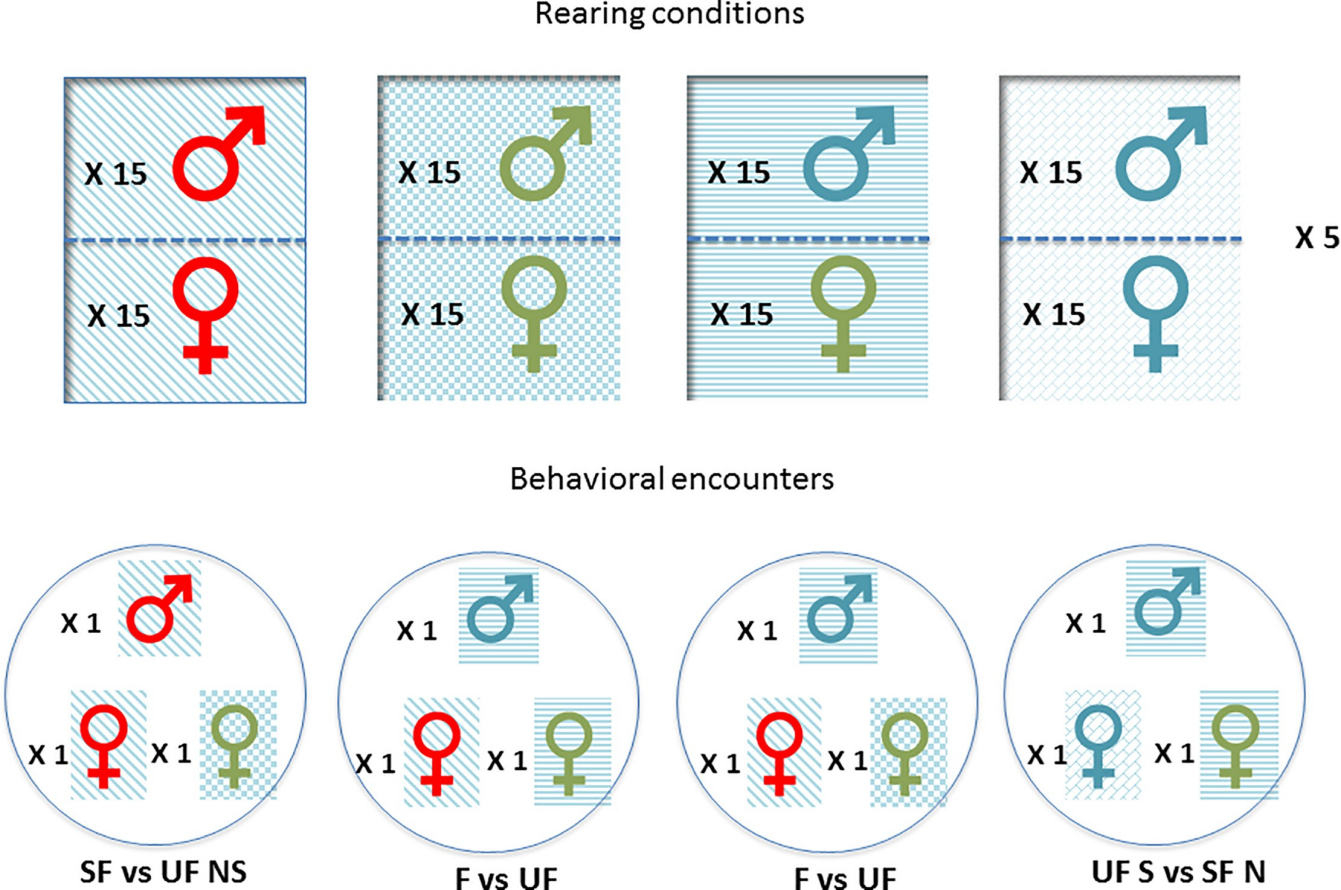
Schematic view of rearing conditions and tested interactions. Familiar individuals were reared in the same box divided in two equal parts with mesh. Individuals of the same color are siblings (same mother). The arrows represent the different types of encounters that were tested in the Y preference and open-field choice tests. Both tested females were either *Wolbachia*-free or *Wolbachia*-infected. F: familiar female; UF unfamiliar female; S: sibling female; NS: nonsibling female; SF: familiar sibling female; UF NS: unfamiliar nonsibling female.

We compared male interactions with familiar and unfamiliar females for both *Wolbachia-*free and *Wolbachia-*infected females and with sibling and nonsibling females for both *Wolbachia-*free and *Wolbachia-*infected females. Finally, we compared the interactions of males with familiar sibling females and unfamiliar nonsibling females for both *Wolbachia-*free and *Wolbachia-*infected females (Fig 2).

### Statistical analysis

Data collected from the choice tests were analyzed using R (Version 3.1.2) [47]. A nonparametric Wilcoxon test was used to compare the time spent by males near each female during the Y preference tests and to compare the number of copulation attempts and their durations during the open-field tests. The proportions of the females’ various reactions to copulation attempts and the proportions of mated females were compared between different kinds of females using Fisher’s exact test. Finally, a Mann-Whitney U test was used to compare the number of offspring between the different mating pairs. The level of statistical significance was set at p≤0.05, and the level of tendency, at p<0.07.

#### Statement about ethical treatment

Within the context of Directive 2010/63/EU on the protection of animals used for scientific purposes, the European Commission decided that most invertebrates, including *A*. *vulgare* (Crustacea: Isopoda), are excluded from ethical considerations. However, we took numerous precautions during our study. The animals we used did not come from the field but from rearing in our lab. All individuals used for the study were reared in groups and were provided with food *ad libitum*. The behavioral experiments were not stressful or invasive. After the behavioral experiments, the animals were reared in large boxes in groups.

## Results

### Female attractiveness: Y preference test

**Fig 3:**
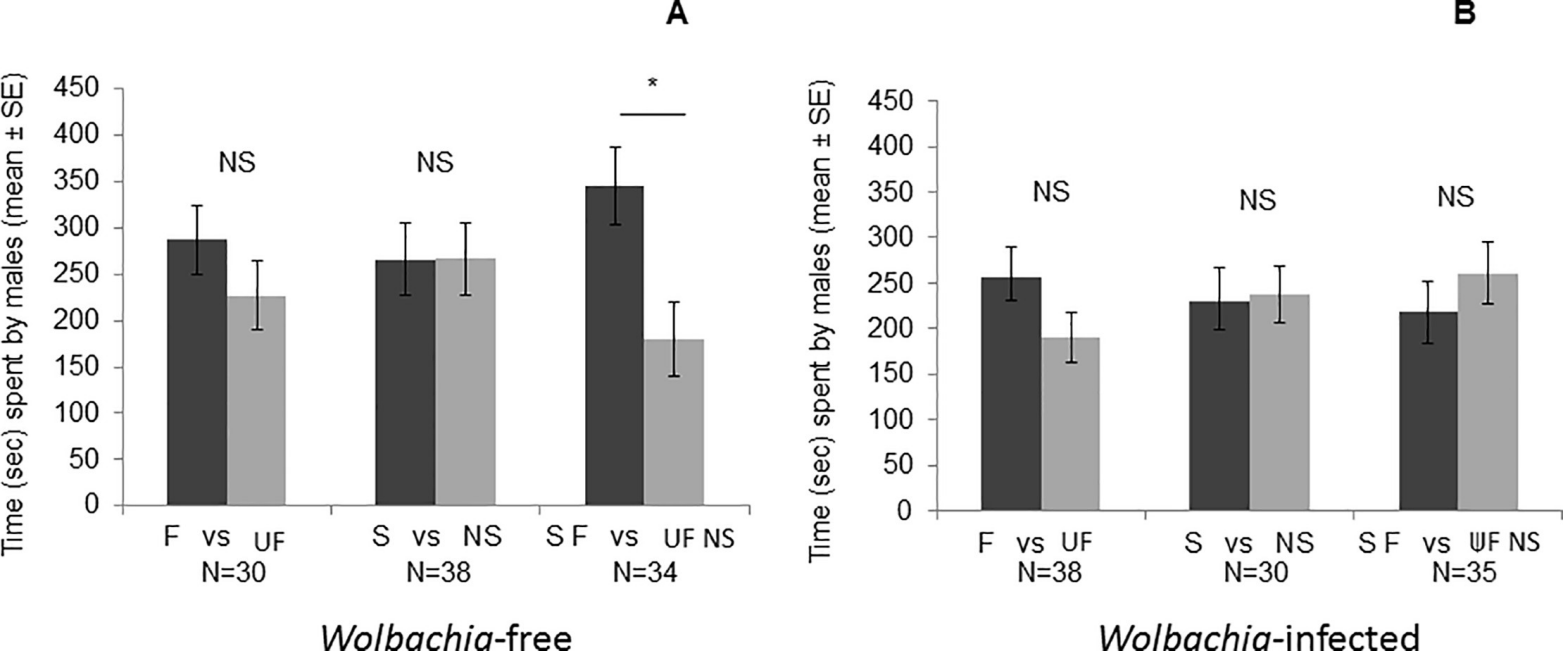
**Time spent by males in the right (RS) and left (LS) sections depending on the presence of familiar or unfamiliar females (F *vs* UF), sibling or nonsibling females (S *vs* NS), and familiar sibling or unfamiliar nonsibling females (SF *vs* UF NS) that were *Wolbachia-*free (A) or *Wolbachia-*infected (B).** Wilcoxon test, *: p≤0.05; NS: p>0.05.

During the Y preference tests, when both females were *Wolbachia-*free, male preferences corresponding to the time spent in front of the section adjacent to females did not differ significantly between familiar and unfamiliar females (Wilcoxon test: Z = 0.73, N = 30, p = 0.46, Fig 3A) nor between sibling and nonsibling females (Wilcoxon test: Z = 0, N = 38, p = 0.99, Fig 3A). Males spent significantly more time in front of the section adjacent to the familiar sibling females than in front of the one adjacent to the unfamiliar nonsibling females (Wilcoxon test: Z = 2.11, N = 34, p = 0.03, Fig 3A). Moreover, they also spend more time in front of the section adjacent to the familiar nonsibling females than in front of that adjacent to the unfamiliar sibling females (437±71sec and 56±34sec respectively; Wilcoxon test: Z = 1.99, N = 6, p = 0.04).

When both females were *Wolbachia-*infected, male preferences did not differ significantly between familiar and unfamiliar females (Wilcoxon test: Z = 0.99, N = 38, p = 0.32, Fig 3B), between sibling and nonsibling females (Wilcoxon test: Z = 0.11, N = 30, p = 0.90, Fig 3B) nor between familiar sibling females and unfamiliar nonsibling females (Wilcoxon test: Z = 0.63, N = 35, p = 0.52, Fig 3B) but did differ significantly between these two groups when both females were *Wolbachia*-free. Finally, we did not notice any difference in the attractiveness between familiar nonsibling females and unfamiliar sibling females when they were *Wolbachia*-infected (176±73sec and 265±84sec; Wilcoxon test: Z = 0.88, N = 10, p = 0.37).

### Interactions between individuals: Open-field choice tests copulation attempts

The number of copulation attempts including all encounters (N = 46 with *Wolbachia*-free females and N = 43 with *Wolbachia-*infected females) was significantly higher when males were tested with two *Wolbachia*-infected females than when they were tested with two *Wolbachia*-free females (1.93±0.18 for 74 attempts and 2.66±0.25 for 68 attempts, respectively; t = -2.34; df = 140; p = 0.02).

**Fig 4:**
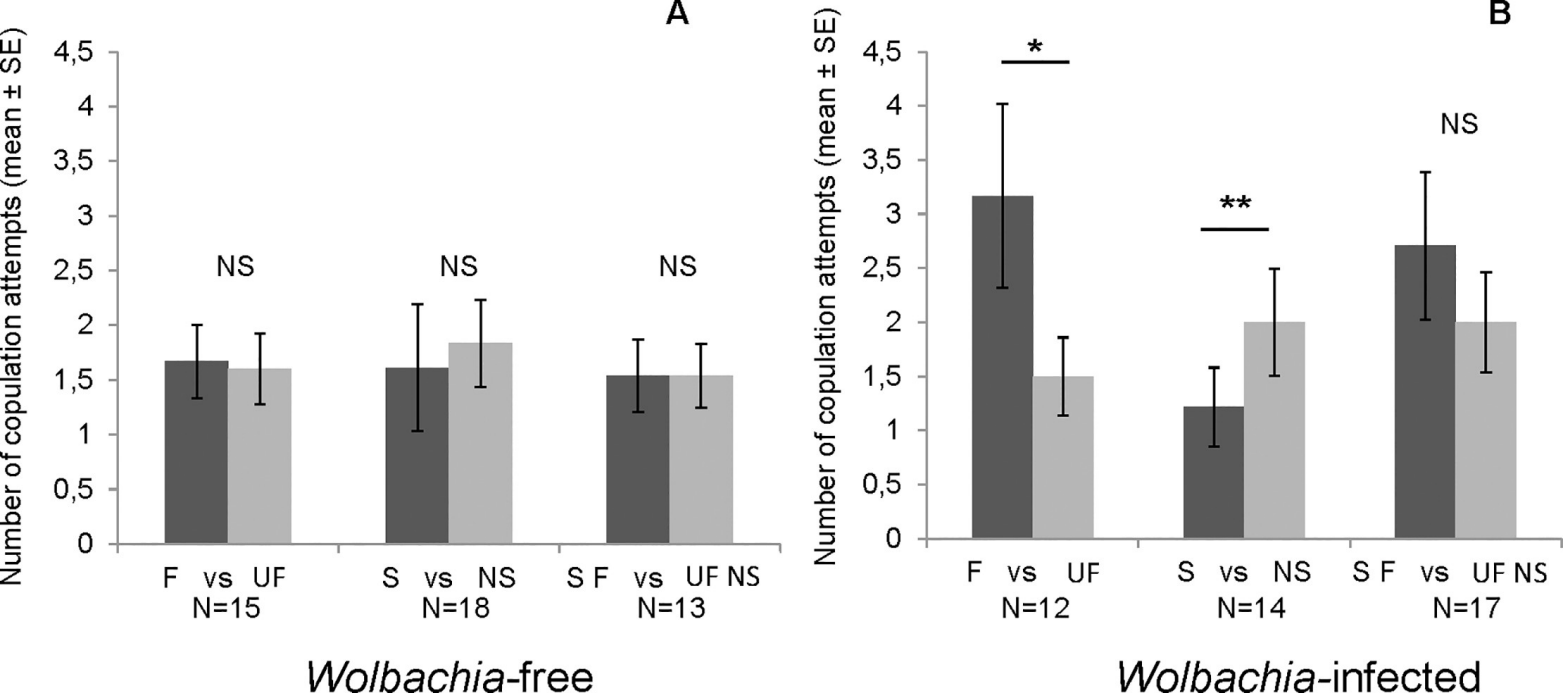
**Number of copulation attempts, from the beginning of the open-field test until the first copulation, with familiar or unfamiliar females (F *vs* UF), sibling or nonsibling females (S *vs* NS), and familiar sibling or unfamiliar nonsibling females (SF *vs* UF NS) that were *Wolbachia-*free (A) or *Wolbachia-*infected (B)**. Wilcoxon test, *: p≤0.05; **: p≤0.01; NS: p>0.05.

When both females were *Wolbachia-*free, the number of copulation attempts did not differ significantly between the two females when males had the choice between familiar and unfamiliar females (Wilcoxon test: Z = 0.03, N = 15, p = 0.97, Fig 4A), sibling and nonsibling females (Wilcoxon test: Z = 1.19, N = 18, p = 0.11, Fig 4A), or familiar sibling and unfamiliar nonsibling females (Wilcoxon test: Z = 0, N = 13, p = 1, Fig 4A).

When both females were *Wolbachia-*infected, males made significantly more copulation attempts with familiar females than with unfamiliar females (Wilcoxon test: Z = 1.82, N = 12, p = 0.034, Fig 4B) and with nonsibling females than with sibling females (Wilcoxon test: Z = 2.34, N = 14, p<0.01, Fig 4B). Finally, the number of copulation attempts did not differ significantly between familiar sibling females and unfamiliar nonsibling females (Wilcoxon test: Z = 1.12, N = 17, p = 0.13, Fig 4B).

The duration of copulation attempts including all encounters (N = 46 with *Wolbachia*-free females and N = 43 with *Wolbachia-*infected females) was significantly longer when males were tested with two *Wolbachia*-free females than when they were tested with two *Wolbachia*-infected females (3690±542 sec for 74 attempts and 1454±305 sec for 68 attempts, respectively; t = -2.34; Df = 140; p = 0.0006).

**Fig 5:**
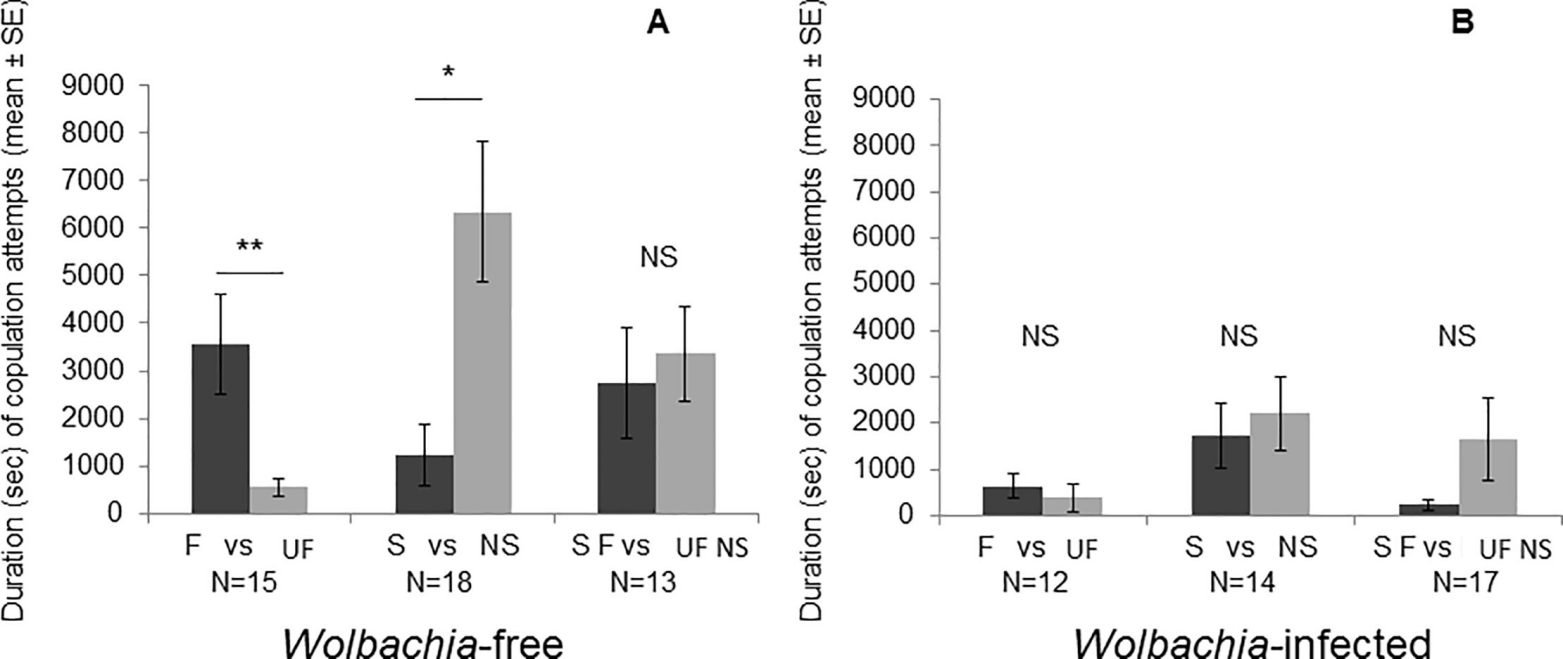
**Duration of copulation attempts, from the beginning of the open-field test until the first copulation, with familiar or unfamiliar females (F *vs* UF), sibling or nonsibling females (S *vs* NS), and familiar sibling females or unfamiliar nonsibling females (SF *vs* UF NS) that were *Wolbachia-*free (A) or *Wolbachia-*infected (B)**. Wilcoxon test, *: p≤0.05; **: p≤0.01; NS: p>0.05.

When both females were *Wolbachia-*free, the duration of male copulation attempts was longer with familiar females than with unfamiliar females (Wilcoxon test: Z = 2.32, N = 15, p = 0.001, Fig 5A) and with nonsibling females than with sibling females (Wilcoxon test: Z = 2.02, N = 18, p = 0.002, Fig 5A). Finally, the duration of copulation attempts did not differ significantly between familiar sibling females and unfamiliar nonsibling females (Wilcoxon test: Z = 1.22, N = 13, p = 0.11, Fig 5A).

When both females were *Wolbachia-*infected, the duration of male copulation attempts did not differ significantly between familiar females and unfamiliar females (Wilcoxon test: Z = 1.25, N = 12, p = 0.1, Fig 5B), nonsibling females and sibling females (Wilcoxon test: Z = 0.21, N = 14, p = 0.41, Fig 5B) nor familiar sibling females and unfamiliar nonsibling females (Wilcoxon test: Z = 0.68, N = 17, p = 0.24, Fig 5B).

Regarding behavioral responses to the copulation attempts, we found that regardless of the infection status of females (*Wolbachia-*free or *Wolbachia-*infected), the proportions of the different behavioral responses did not differ significantly between familiar *vs*. unfamiliar females (Fisher’s exact test for *Wolbachia-*free females: p = 0.62, and for *Wolbachia-*infected females: p = 0.83) or between sibling familiar *vs*. nonsibling unfamiliar females (Fisher’s exact test for *Wolbachia-*free females: p = 1, and p = 0.07; for *Wolbachia-*infected females: p = 0.28,) but tended to differ between sibling *vs*. nonsibling females (Fisher’s exact test for *Wolbachia-*free females: p = 0.07; for *Wolbachia-*infected females: p = 0.02).

The proportion of females that accepted copulation did not differ between the different kinds of females, regardless of infection status (*Wolbachia-*free or *Wolbachia-*infected) (S1 Table).

**Fig 6:**
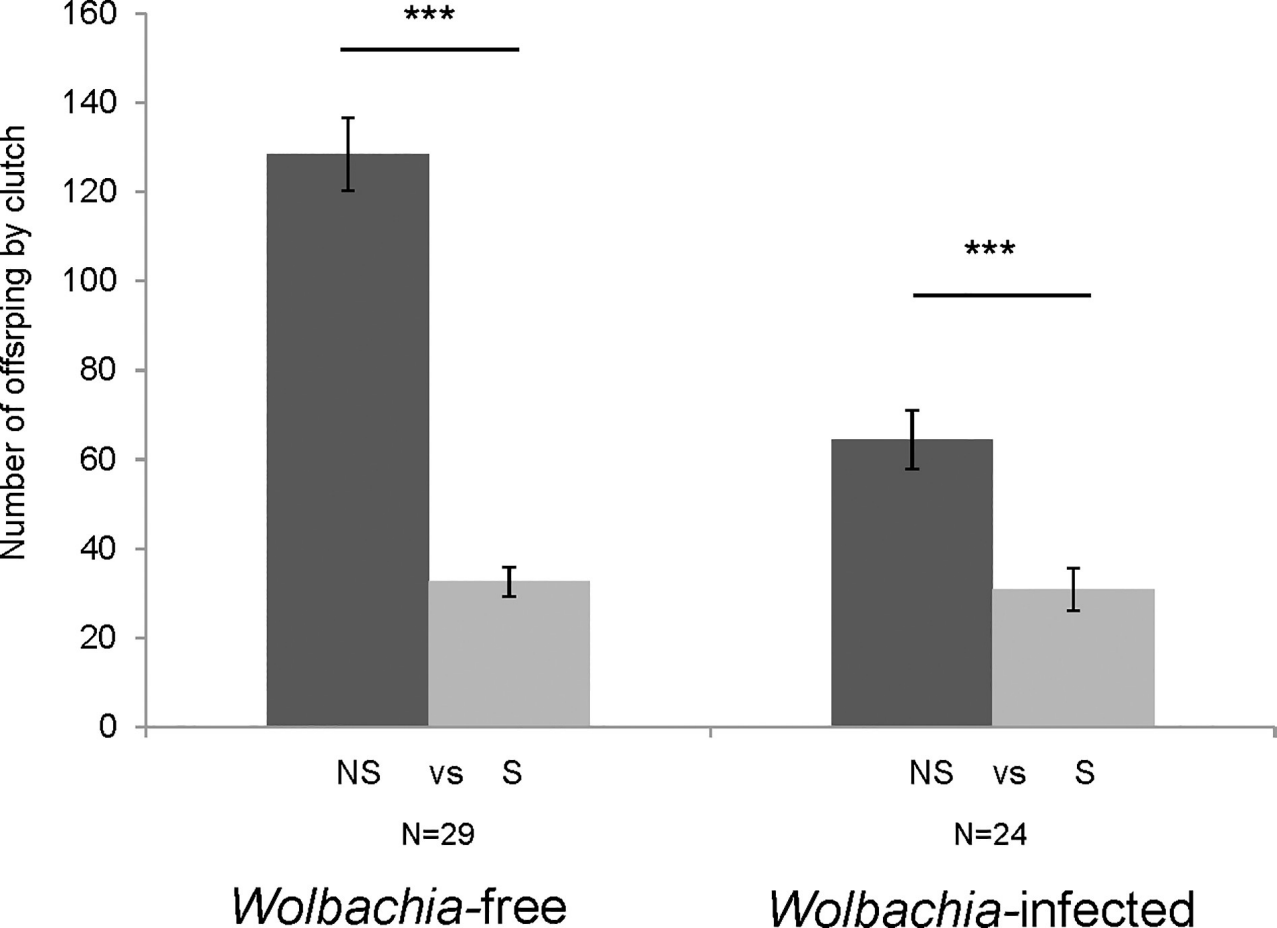
Number of offspring for nonsibling and sibling mates with *Wolbachia-*free females and *Wolbachia-*infected females. Mann-Whitney test, ***: p < 0.001.

The effect of relatedness between mates on female fertility was measured using offspring number. Sibling mates had fewer offspring than nonsibling mates for both *Wolbachia-*free females (Mann-Whitney test: U = 18, N = 29, p<0.0001, Fig 6) and *Wolbachia-*infected females (Mann-Whitney test: U = 114, N = 24, p = 0.0003, Fig 6). Moreover, in the case of nonsibling mates, *Wolbachia-*free females had more offspring than *Wolbachia-*infected females (Mann-Whitney test: U = 43, N = 24, p<0.0001, Fig 6). In contrast, in the case of sibling mates, there was no significant difference in the number of offspring between *Wolbachia-*free females and *Wolbachia-*infected females (Mann-Whitney test: U = 280, N = 24, p = 0.86, Fig 6).

## Discussion

Our results revealed that males were attracted to all females and showed preferences in only a few cases. In the Y preference tests, males were more attracted to familiar full-sibling females than to unfamiliar nonsibling females and only when those females were *Wolbachia*-free. Indeed, when the males and females could interact directly, the number of copulation attempts was affected by neither relatedness nor familiarity when both females were both *Wolbachia*-free. However, even though the number of copulations was not affected, the duration of copulations attempts was longer for familiar and unrelated females. In contrast, when females were *Wolbachia*-infected, males made more attempts with familiar females and unrelated females, but the duration was significantly different between those types of females. The preference seems to be based on the familiarity of individuals and affected by *Wolbachia* infections. When males mated with a full sibling female, inbreeding depression was indicated by a severe decrease in fecundity (fewer offspring). The results obtained in the Y preference tests underline the complexity of the attractiveness of individuals and the importance of direct contact in the selection of potential mates. A previous study showed that in this species, males showed preferences between two females according to genetic characteristics; specifically, based on their level of genetic similarity, males were able to discriminate the most dissimilar females in Y tests [48].

### In a reproductive context, males prefer nonsibling females to full-sibling females and familiar females to unfamiliar females when both females are *Wolbachia*-free

Behavioral observations indicated that when a male can interact with sibling *vs*. nonsibling females or familiar *vs*. unfamiliar females, both kinship and familiarity influence male mate choice and underlie the male’s capacity to discriminate between two degrees of relatedness.

When males interacted with one sibling female and one nonsibling female, they attempted to copulate longer with the nonsibling female than with the sibling female. This preference for unrelated females may indicate a precopulatory mechanism of inbreeding avoidance in this species. A male's reproductive investment has already been shown to vary based on the infection status of the female, specifically for the number of copulation attempts and copulations [39] and for the quantity of sperm released [49].

Precopulatory mechanisms of inbreeding avoidance have been demonstrated in other species (e.g., the German cockroatch *Blatella germanica* [26], the field cricket *Gryllus bimaculatus* [50], sticklebacks [51], the parasitoid wasp *Habrobracon hebetor* [52], the ant *Iridomyrmex humilis* [53], and the mole rat *Heterocephalus glaber* [54]). For example, when female three-spined sticklebacks (*Gasterosteus aculeatus*) can choose between a familiar full sibling male and an unfamiliar nonsibling male, they prefer to mate with the nonsibling male and can discriminate between the two based on olfactory and visual cues [51]. Kin recognition may be due to familiarity and/or relatedness effects [14]. We tried to disentangle the two types of effects and showed that mechanism of inbreeding avoidance seems to be driven only by relatedness in *A*. *vulgare*. Indeed, the courting duration was longer between unrelated males and females than between related ones and between familiar males and females than between unfamiliar ones. The recognition of siblings based on genetic cues occurs in several species (e.g., the ground squirrel *Urocitellus beldingi* [13, 26], the ant *Iridomyrmex humilis* [53], the mouse *Mus musculus* [55], the German cockroatch *Blatella germanica* [56], the ladybird *Manochilus sexmaculatus* [57], *Drosophila melanogaster* [58], the Japanese quail [59], and the zebra finch *Taeniopygia guttata* [60]). Female field crickets try to escape and fight back more when they are courted by full siblings than when they are courted by unrelated males, even if they have no prior experience of conspecifics, suggesting discrimination based on relatedness in this species [50]. Males are also more reluctant to court full sibling females than unrelated females [50]. Other experiments in the same species and in closely related species have shown that females also perform postcopulatory mate choice by using more sperm from unrelated males than from related ones [61–63].

The finding that courting duration is longer when males and females are familiar may provide some insight into the behavioral ecology of *A*. *vulgare*. Indeed, we know little about the dispersal ability of this species, but the existence of a strategy to limit inbreeding based on kin recognition suggest that individuals do not necessarily grow and live in families all their lives but may encounter each other at the adult stage, after they have dispersed. A kinship recognition system based on relatedness rather than familiarity would allow individuals to avoid inbreeding even after dispersal. The preference for familiar females may indicate a strategy to avoid outbreeding. Indeed, we can hypothesize that within a population of *A*. *vulgare*, all individuals share the same habitat and are familiar with each other. Familiarity assessment may limit copulations with individuals from another population, thereby decreasing outbreeding. In a few species, mating is apparently random from a genetic point of view [64]. In specific situations, such as when alternative mates are not available, the cost of avoiding inbreeding may be too high, causing mating with close relatives (brothers or sisters) to be favorable, even if such cases are rare in natural populations [65]. Further experiments are needed to better understand the potential costs of outbreeding in this species. Overall, our study indicates that, similar to observations in other species, mechanisms of kinship discrimination occur in *A*. *vulgare*.

### Kinship discrimination allows individuals to decrease inbreeding depression

The evolution of kinship discrimination in a mating context should occur when mating with siblings leads to a decrease in individual fitness. Our study revealed that, in *A*. *vulgare*, mating with full siblings induces a critical decline in reproductive success. This decrease in the number of offspring could be due to the decreased sperm allocation of males [66], a phenomenon that has been observed when *A*. *vulgare* males mate with *Wolbachia-*infected females [49]. However, this decrease in sperm allocation does not lead to a decrease in fertility unless the male fertilizes a large number of females [49].

The decrease in offspring observed here revealed that mating with full siblings leads to inbreeding depression in *A*. *vulgare*, which appears in the first generation. A decrease in fertility due to inbreeding depression has been observed in many species [26, 67–70]. Our study reveals that in *A*. *vulgare*, one generation of inbreeding is sufficient to lead to a decrease in fertility, as observed in *Blattella germanica* [26]. In other species, the deleterious effects of inbreeding may appear later. In the bulb mite, inbreeding depression in terms of fecundity appears after only one generation, and that in terms of survival and sterility increases after 6 generations [71]. In *A*. *vulgare*, further experiments are needed to investigate the long-term effect of inbreeding on the fitness of offspring. Indeed, in several species, harmful effects of inbreeding depression impact offspring fitness in particular [2], for example, via a lower growth rate and lower fecundity [72], lower sperm competition success [73], a decrease in attractiveness to mates [74], a higher parasitism risk [75] and a higher mortality risk [71]. To conclude, the mating preference of *A*. *vulgare* males for nonsibling females leads to greater fitness. However, we observed no behavioral mechanism that totally prevented sibling mating; instead, we observed differential investment in terms of the number or duration of copulation attempts in favor of nonsibling females.

### Attractiveness between individuals depends on the context

The behavior of males observed in a mating context revealed a preference for nonsibling females (in terms of either copulation number or duration), a mechanism contributing to a decrease in inbreeding. However, the results obtained in the Y preference tests indicated a preference of males for familiar sibling females. While Y preference tests provide some information about social preferences, they may not be sufficient for investigating mate choice. Thus, it is important to perform behavioral choice tests under conditions that are as close as possible to natural ones by letting animals interact and express mating behavior. Our results are in good agreement with those from a previous study on *B*. *germanica*, which showed that individuals' preferences were context dependent [56]. Cockroaches preferred nonsibling individuals in a mating context and sibling individuals in a social context. The authors hypothesized that by choosing siblings as social partners, individuals increased their inclusive fitness through the advantages of grouping. We can form the same hypothesis for *A*. *vulgare*, in which gregariousness provides many benefits [25], such as the limitation of water loss [76], an increase in body growth [77] and a faster onset of reproduction [78, 79]. In addition, it may have been more difficult for the tested males to discriminate, without bodily contact, the relatedness of the familiar females. Open-field tests provided males with the opportunity to interact with females and express mating behavior and provided females with the opportunity to react to the male’s copulation attempts by accepting or refusing the copulation. In contrast, the Y preference tests seemed to reveal social preferences in a context where discrimination may have been more difficult than in the open-field choice tests, especially when females were *Wolbachia-*infected.

### Male mating preferences for familiar females and for nonsibling females are also found with *Wolbachia*-infected females, but social preferences are modulated

The number of copulation attempts was not significantly impacted by familiarity nor relatedness but the duration of copulation attempts was longer with familiar females and with nonsibling females when males were tested with two *Wolbachia*-free females. However, the number of copulation attempts was higher with familiar females and with nonsibling females but the duration of copulation attempts was not impacted by familiarity nor relatedness when males were tested with two *Wolbachia*-infected females. In both situations, we observed a preference for familiar females and for nonsibling females but at different stages of the courtship. The mating strategy was different according to female infection status. The overall number of attempts was lower but the duration was higher with *Wolbachia*-free females than with *Wolbachia*-infected females. *Wolbachia* infection is known to modulate females' attractiveness. Indeed, when males and females can interact in a mating context, males prefer *Wolbachia-*free females over *Wolbachia-*infected females [39]. This preference may be an adaptive response to the cost related to *Wolbachia* infection. *Wolbachia-*infected females have decreased fitness due to the effect of *Wolbachia* on the immune system [38], growth [80], fertility [81] and cognition [37]. Moreover, the current study also indicate that *Wolbachia* decrease the number of descendants of infected females. *Wolbachia-*infected females, like *Wolbachia-*free females, also suffer a decrease in fertility when they mate with sibling males, indicating that the infection does not decrease the cost of inbreeding depression. When males could interact with a sibling female and a nonsibling female, both of which were *Wolbachia-*infected, males performed more copulation attempts with the nonsibling female. This result indicated that the presence of *Wolbachia* did not impair the males’ discrimination of females with different degrees of relatedness. The results obtained in the Y test for *Wolbachia-*infected females revealed the absence of male preference at a short distance. Indeed, males did not express any social preference when both females were infected with *Wolbachia*. However, when both females were *Wolbachia*-free, males showed preferences for both sibling females and familiar females. A possible explanation for this difference is that by modifying the odor of its host [27, 41], *Wolbachia* may impede the ability of males to discriminate other characteristics of females, such as their familiarity or their relatedness. When individuals are in direct contact, the observed differences (number or duration of copulation attempts) can be linked to female behavior in reaction to males’ solicitations. As explained before, *Wolbachia* are known to affect their host’s behavior, and the main differences observed here could be the results of infected females’ behavioral profiles.

To conclude, the present study shows that mate choice can exists in *A*. *vulgare*, as male individuals are able to discriminate, in some specific cases, between females depending on familiarity and kinship. In this species, kin recognition appears to be essential for avoiding the high costs of inbreeding depression. When females are *Wolbachia-*infected, social preferences based on odors are modified [41]. Further studies are needed to better understand the preference for familiar females and the potential costs of outbreeding in this species and why familiar sibling females are more attractive than unfamiliar nonsibling females in choice tests with no consequence in terms of copulation attempts.

## Supporting information

S1 Dataset Raw data of the current study. (XLSX)

S1 Table Number of familiar (F) and unfamiliar (NF), sibling (S) and non-sibling (NS), and sibling familiar (SF) and non-sibling unfamiliar (NS NF) females that accepted and refused the copulation attempts during the open-field tests, according to their infection status (W-: *Wolbachia-*free; W+: *Wolbachia-*infected). Comparison of females copulation (accepted vs refused) distribution was tested using the Exact Fisher test.
(DOCX)

S1 Video Video of *Armadillidium vulgare* male and female copulation attempt: Volvation immediately followed by a slight opening, indicating that mating was accepted by the female. (MP4)

S2 Video Video of *Armadillidium vulgare* male and female copulation attempt: Rolling without opening: Volvation that did not allow the male to copulate with the female. (MP4)

## References

[1] Andersson MB. Sexual selection: Princeton University Press; 1994.

[2] Keller LF, Waller DM. Inbreeding effects in wild populations. Trends Ecol Evol. 2002;17(5):230–41.

[3] Charlesworth D, Willis JH. The genetics of inbreeding depression. Nature Reviews Genetics. 2009;10(11):783–96. 10.1038/nrg2664 19834483

[4] Slate J, Kruuk L, Marshall T, Pemberton J, Clutton-Brock T. Inbreeding depression influences lifetime breeding success in a wild population of red deer (*Cervus elaphus*). Proc R Soc B. 2000;267(1453):1657–62. 10.1098/rspb.2000.1192 11467429 PMC1690715

[5] Höglund J, Piertney SB, Alatalo RV, Lindell J, Lundberg A, Rintamäki PT. Inbreeding depression and male fitness in black grouse. Proc R Soc B. 2002;269(1492):711–5. 10.1098/rspb.2001.1937 11934362 PMC1690954

[6] Liu X, Tu X, He H, Chen C, Xue F. Evidence for inbreeding depression and pre-copulatory, but not post copulatory inbreeding avoidance in the cabbage beetle *Colaphellus bowringi*. Plos One. 2014;9(4):e94389 10.1371/journal.pone.0094389 24718627 PMC3981785

[7] Vayssade C, De Fazio Cl, Quaglietti B, Auguste A, Ris N, Fauvergue X. Inbreeding depression in a parasitoid wasp with single-locus complementary sex determination. Plos One. 2014;9(6):e97733 10.1371/journal.pone.0097733 24892828 PMC4043504

[8] Lebigre C, Alatalo RV, Siitari H. Female-biased dispersal alone can reduce the occurrence of inbreeding in black grouse (*Tetrao tetrix*). Mol Ecol. 2010;19(9):1929–39. 10.1111/j.1365-294X.2010.04614.x 20345672

[9] Szulkin M, Sheldon BC. Dispersal as a means of inbreeding avoidance in a wild bird population. Proc Roy Soc B. 2008;275(1635):703–11. 10.1098/rspb.2007.0989 PMC2596843 18211876

[10] Lihoreau M, Rivault C. German cockroach males maximize their inclusive fitness by avoiding mating with kin. Anim Behav. 2010;80(2):303–9.

[11] Pusey A, Wolf M. Inbreeding avoidance in animals. Trends Ecol Evol. 1996;11(5):201–6. 21237809 10.1016/0169-5347(96)10028-8

[12] Holmes WG, Sherman PW. Kin recognition in animals: The prevalence of nepotism among animals raises basic questions about how and why they distinguish relatives from unrelated individuals. Am Scientist. 1983;71(1):46–55.

[13] Mateo JM. Self-referent phenotype matching and long-term maintenance of kin recognition. Animal Behaviour. 2010;80(5):929–35.

[14] Mateo JM. Recognition systems and biological organization: The perception component of social recognition. Ann Zool Fenn. 2004;41(6):729–45.

[15] Richard FJ, Hunt JH. Intracolony chemical communication in social insects. Insect Soc. 2013;60(3):275–91.

[16] Lacy RC, Sherman PW. Kin recognition by phenotype matching. Am Nat. 1983:489–512.

[17] Blaustein AR. Kin recognition mechanisms: phenotypic matching or recognition alleles? Am Nat. 1983;121(5):749–54.

[18] Ross KG, Keller L. Genetic control of social organization in a ant. Proc Natl Acad Sci. 1998;95:14232–7. 9826683 10.1073/pnas.95.24.14232 PMC24356

[19] Parker GA. Sexual conflict over mating and fertilization: an overview. Philosophical Transactions of the Royal Society B: Biological Sciences. 2006;361(1466):235–59. 10.1098/rstb.2005.1785 PMC1569603 16612884

[20] Metzger M, Bernstein C, Hoffmeister TS, Desouhant E. Does kin recognition and sib-mating avoidance limit the risk of genetic incompatibility in a parasitic wasp? Plos One. 2010;5(10):e13505 10.1371/journal.pone.0013505 20976063 PMC2957437

[21] Reynolds SM, Uy JAC, Patricelli GL, Coleman SW, Braun MJ, Borgia G. Tests of the kin selection model of mate choice and inbreeding avoidance in satin bowerbirds. Behav Ecol. 2014;25(4):1005–14.

[22] Ala‐Honkola O, Manier MK, Lüpold S, Pitnick S. No evidence for postcopulatory inbreeding avoidance in *Drosophila melanogaster*. Evolution. 2011;65(9):2699–705. 10.1111/j.1558-5646.2011.01317.x 21884066

[23] Huang MH, Caillaud MC. Inbreeding avoidance by recognition of close kin in the pea aphid, *Acyrthosiphon pisum*. J Insect Sci. 2012;12(1):39. 22954315 10.1673/031.012.3901 PMC3471804

[24] Beauché F, Richard F-J. The best timing of mate search in *Armadillidium vulgare* (Isopoda, Oniscidea). Plos One. 2013;8(3):e57737 10.1371/journal.pone.0057737 23469225 PMC3585876

[25] Broly P, Deneubourg JL, Devigne C. Benefits of aggregation in woodlice: a factor in the terrestrialization process? Insect Soc. 2013;60(4):419–35.

[26] Lihoreau M, Zimmer C, Rivault C. Kin recognition and incest avoidance in a group-living insect. Behav Ecol. 2007;18(5):880–7.

[27] Fortin M, Debenest C, Souty-Grosset C, Richard F-J. Males prefer virgin females, even if parasitized, in the terrestrial isopod Armadillidium vulgare. Ecol Evol. 2018; 10.1002/ece3.3858 29607029 PMC5869267

[28] Beltran-Bech S, Richard F-J. Impact of infection on mate choice. Animal Behaviour. 2014;90:159–70.

[29] Werren JH, Windsor D, Guo LR. Distribution of *Wolbachia* among neotropical arthropods. Proc Roy Soc B. 1995;262(1364):197–204.

[30] Sironi M, Bandi C, Sacchi L, DiSacco B, Damiani G, Genchi C. Molecular evidence for a close relative of the arthropod endosymbiont *Wolbachia* in a filarial worm. Molecular and Biochemical Parasitology. 1995;74(2):223–7. 8719164 10.1016/0166-6851(95)02494-8

[31] Baldo L, Prendini L, Corthals A, Werren JH. *Wolbachia* are present in Southern African scorpions and cluster with supergroup F. Curr Microbiol. 2007;55(5):367–73. 10.1007/s00284-007-9009-4 17676427

[32] Bouchon D, Rigaud T, Juchault P. Evidence for widespread *Wolbachia* infection in isopod crustaceans: molecular identification and host feminization. Proc Roy Soc B. 1998;265(1401):1081–90. 10.1098/rspb.1998.0402 PMC1689171 9684374

[33] Werren JH, Baldo L, Clark ME. *Wolbachia*: master manipulators of invertebrate biology. Nature Reviews Microbiology. 2008;6(10):741–51. 10.1038/nrmicro1969 18794912

[34] Martin G, Juchault P, Legrand J. Mise en évidence d'un micro-organisme intracytoplasmique symbiote de l'oniscoïde *Armadillidium vulgare* Latr. dont la présence accompagne l'intersexualité ou la féminisation totale des mâles génétiques de la lignée thélygène. Comptes rendus de l'Académie des Sciences de Paris. 1973;276:2313–6.

[35] Juchault P, Rigaud T, Mocquard JP. Evolution of sex-determining mechanisms in a wild population of *Armadillidium vulgare* Latr.(Crustacea, Isopoda): competition between two feminizing parasitic sex factors. Heredity. 1992;69:382–90.

[36] Le Clec'h W, Dittmer J, Raimond M, Bouchon D, Sicard M. Phenotypic shift in *Wolbachia* virulence towards its native host across serial horizontal passages. Proc Roy Soc B. 2017;284 20171076. 10.1098/rspb.2017.1076 PMC5543228 28724736

[37] Templé N, Richard F-J. Intra-cellular bacterial infections affect learning and memory capacities of an invertebrate. Frontiers in zoology. 2015;12(1):1 10.1186/s12983-014-0093-6 26675213 PMC4678612

[38] Braquart-Varnier C, Lachat M, Herbiniere J, Johnson M, Caubet Y, Bouchon D, et al *Wolbachia* mediate variation of host immunocompetence. Plos One. 2008;3(9). 10.1371/journal.pone.0003286 PMC2546445 18818770

[39] Moreau J, Bertin A, Caubet Y, Rigaud T. Sexual selection in an isopod with *Wolbachia*-induced sex reversal: males prefer real females. Journal of Evolutionary Biology. 2001;14(3):388–94.

[40] Moreau J, Bertin A, Caubet Y, Rigaud T. Sexual selection in an isopod with *Wolbachia*- induced sex reversal: males prefer real females. Journal of Evolutionary Biology. 2001;14:388–94.

[41] Richard F-J. Symbiotic bacteria influence the odor and mating preference of their hosts. Frontiers in Ecology and Evolution. 2017;5:143.

[42] Cordaux R, Michel-Salzat A, Frelon-Raimond M, Rigaud T, Bouchon D. Evidence for a new feminizing Wolbachia strain in the isopod *Armadillidium vulgare*: evolutionary implications. Heredity. 2004;93:78–84. 10.1038/sj.hdy.6800482 15138452

[43] Moreau J, Rigaud T. Operational sex ratio in terrestrial isopods: interaction between potential rate of reproduction and *Wolbachia-*induced sex ratio distortion. Oikos. 2000;91(3):477–84.

[44] Steel C. Mechanisms of coordination between moulting and reproduction in terrestrial isopod Crustacea. The Biological Bulletin. 1980;159(1):206–18.

[45] Moreau J, Rigaud T. The shape of calcium carbonate deposits as an external marker for female reproductive status in terrestrial isopods. Journal of Crustacean Biology. 2002;22(2):353–6.

[46] Mead F. Recherches sur la reproduction et le comportement sexuel des Isopodes terrestres. Unpublished PhD Dissertation Université de Provence, France. 1973.

[47] Team RDC. R: A Language and Environment for Statistical Computing R Foundation for Statistical Computing, Vienna, Austria; 2008.

[48] Durand S, Beauché F, Richard FJ, Beltran‐Bech S. How do females’ genetic characteristics influence male mate preference in the terrestrial isopod *Armadillidium vulgare*? Ethology. 2015;121(11):1122–30.

[49] Rigaud T, Moreau M. A cost of *Wolbachia*-induced sex reversal and female-biased sex ratios: decrease in female fertility after sperm depletion in a terrestrial isopod. Proc Roy Soc B. 2004;271(1551):1941–6. 10.1098/rspb.2004.2804 PMC1691816 15347518

[50] Simmons LW. Kin recognition and its influence of mating preferences of the field cricket, *Gryllus bimaculatus* (Degeer). Anim Behav. 1989;38:68–77.

[51] Frommen JG, Bakker TCM. Inbreeding avoidance through non-random mating in sticklebacks. Biological Letters. 2006;2(2):232–5. 10.1098/rsbl.2005.0432 PMC1618905 17148370

[52] Ode PJ, Antolin MF, Strand MR. Brood-mate avoidance in the parasitic wasp *Bracon hebetor say*. Animal Behaviour. 1995;49(5):1239–48.

[53] Keller L, Passera L. Incest avoidance, fluctuating asymetry, and the consequences of inbreeding in *Iridomyrmex humilis*, an ant with multiple queen colonies. Behav Ecol Sociobiol. 1993;33(3):191–9.

[54] Clarke F, Faulkes C. Kin discrimination and female mate choice in the naked mole-rat *Heterocephalus glaber*. Proc R Soc B. 1999;266(1432):1995–2002. 10.1098/rspb.1999.0877 10584337 PMC1690316

[55] Barnard CJ, Fitzsimons J. Kin recognition and mate choice in mice- The effects of kinship, familiarity and social interference on intersexual interaction. Animal Behaviour. 1988;36:1078–90.

[56] Lihoreau M, Rivault C. Kin recognition via cuticular hydrocarbons shapes cockroach social life. Behav Ecol. 2009;20(1):46–53.

[57] Saxena S, Mishra G, Omkar. Inbreeding avoidance in aphidophagous ladybird beetles: a case study in *Menochilus sexmaculatus*. Can J Zool. 2016;94(5):361–5.

[58] Loyau A, Cornuau JH, Clobert J, Danchin E. Incestuous sisters: mate preference for brothers over unrelated males in *Drosophila melanogaster*. Plos One. 2012;7(12). 10.1371/journal.pone.0051293 PMC3519633 23251487

[59] Bateson P. Preferences for cousins in Japanese quail. Nature. 1982;295(5846):236–7.

[60] Burley N, Minor C, Strachan C. Social preference of Zebra finches for siblings, cousins and non-kin. Anim Behav. 1990;39:775–84.

[61] Tuni C, Beveridge M, Simmons LW. Female crickets assess relatedness during mate guarding and bias storage of sperm towards unrelated males. J Evol Biol. 2013;26(6):1261–8. 10.1111/jeb.12118 23745826

[62] Stockley P. Sperm selection and genetic incompatibility: does relatedness of mates affect male success in sperm competition? Proc R Soc B. 1999;266(1429):1663–9.

[63] Bretman A, Wedell N, Tregenza T. Molecular evidence of post-copulatory inbreeding avoidance in the field cricket *Gryllus bimaculatus*. Proc R Soc B. 2004;271(1535):159–64. 10.1098/rspb.2003.2563 15058392 PMC1691572

[64] Keane B, Creel SR, Waser PM. No evidence of inbreeding avoidance or inbreeding depression in a social carnivore. Behav Ecol. 1996;7:480–9.

[65] Thornhill NW. The natural history of inbreeding and outbreeding Chicago: University of Chicago Press; 1993.

[66] Wedell N, Gage MJG, Parker GA. Sperm competition, male prudence and sperm-limited females. Trends in Ecology & Evolution. 2002;17(7):313–20.

[67] Lihoreau M, Zimmer C, Rivault C. Mutual mate choice: when it pays both sexes to avoid inbreeding. Plos One. 2008;3(10):e3365 10.1371/journal.pone.0003365 18843373 PMC2557063

[68] Pitcher TE, Rodd FH, Rowe L. Female choice and the relatedness of mates in the guppy (*Poecilia reticulata*). Genetica. 2008;134(1):137–46. 10.1007/s10709-008-9246-x 18297404

[69] Tregenza T, Wedell N. Polyandrous females avoid costs of inbreeding. Nature. 2002;415(6867):71–3. 10.1038/415071a 11780118

[70] Zhang B, Xue HJ, Song KQ, Liu J, Li WZ, Nie RE, et al Male mate recognition via cuticular hydrocarbons facilitates sexual isolation between sympatric leaf beetle sister species. J Insect Physiol. 2014;70:15–21. 10.1016/j.jinsphys.2014.08.006 25172230

[71] Radwan J. Inbreeding depression in fecundity and inbred line extinction in the bulb mite, *Rhizoglyphus robini*. Heredity. 2003;90(5):371–6. 10.1038/sj.hdy.6800254 12714982

[72] Roff DA. Effects of inbreeding on morphological and life history traits of the sand cricket, *Gryllus firmus*. Heredity. 1998;81:28–37.

[73] Konior M, Keller L, Radwan J. Effect of inbreeding and heritability of sperm competition success in the bulb mite *Rhizoglyphus robini*. Heredity. 2005;94(6):577–81. 10.1038/sj.hdy.6800649 15742000

[74] McKee AA, Newton SM, Carter AJR. Influence of inbreeding on female mate choice in two species of *Drosophila*. J Insect Behav. 2014;27(5):613–25. 10.1007/s10905-014-9453-5 29225418 PMC5718203

[75] Coltman DW, Pilkington JG, Smith JA, Pemberton JM. Parasite-mediated selection against inbred Soay sheep in a free-living, island population. Evolution. 1999;53(4):1259–67. 10.1111/j.1558-5646.1999.tb04538.x 28565537

[76] Allee WC. Studies in animal aggregations: Causes and effects of bunching in land isopods. J Exp Zool. 1926;45(1):255–77.

[77] Takeda N. The aggregation pheromone of some terrestrial isopod crustaceans. Experientia. 1980;36(11):1296–7.

[78] Lefebvre F, Caubet Y. On the male-effect in the terrestrial Crustacean *Armadillidium vulgare* (Latreille, 1804). Invertebr Reprod Dev. 1999;35(1):55–64.

[79] Lefebvre F, Caubet Y. Female-extended control over their reproductive investment: the role of early mating interactions on oocyte maturation in the terrestrial crustacean *Armadillidium vulgare* (Latreille, 1804). Invertebr Reprod Dev. 2010;54(4):177–86.

[80] Juchault P, Mocquard J. Effetcs of inoculation with feminizing endocellular bacteria on growth and reproduction in females of the oniscoid crustacean *Armadillidium vulgare* (Latr)- Possible impact on the evolution of populations. Crustaceana. 1989;56:83–92.

[81] Moreau J, Seguin S, Caubet Y, Rigaud T. Female remating and sperm competition patterns in a terrestrial crustacean. Animal Behaviour. 2002;64:569–77.

