# Supplementary material for: How do familiarity and relatedness influence mate choice in Armadillidium vulgare?
Source: PLoS One. 2018 Dec 31;13(12):e0209893. doi: 10.1371/journal.pone.0209893 (PMC6312335; doi:10.1371/journal.pone.0209893)
Supplement: S1 Table — Comparison of females copulation (accepted vs refused) distribution was tested using the Exact Fisher test. (DOCX) [file pone.0209893.s002.docx]

**Supplementary Table. 1.** Number of familiar (F) and unfamiliar (UF), sibling (S) and nonsibling (NS), and familiar sibling (FS) and unfamiliar nonsibling (UF NS) females that accepted and refused the copulation attempts during the open-field tests, according to their infection status (W- : *Wolbachia-*free; W+: *Wolbachia-*infected). Comparison of female copulation (accepted vs refused) distributions were tested using the Exact Fisher test.

|  | *Wolbachia*-free- | | | | | | Infected with *Wolbachia* | | | | | |
| --- | --- | --- | --- | --- | --- | --- | --- | --- | --- | --- | --- | --- |
|  | F | UF | S | NS | FS | UF NS | F | UF | S | NS | FS | UF NS |
|  | N=15 | | N=18 | | N=13 | | N=12 | | N=14 | | N=17 | |
| Nb of females which accepted the copulation | 1 | 3 | 2 | 5 | 4 | 5 | 2 | 0 | 2 | 5 | 4 | 5 |
| Nb of females which refused the copulation | 14 | 12 | 16 | 13 | 9 | 8 | 10 | 12 | 12 | 9 | 13 | 12 |
| Significance of the Exact Fisher test | 0.59 | | 0.33 | | 0.72 | | 0.47 | | 0.31 | | 0.74 | |
